# Genital and anal injury in women after sexual assault: prevalence rates and associated risk factors in 294 cases

**DOI:** 10.1007/s00414-025-03522-1

**Published:** 2025-05-24

**Authors:** Daniel Kane, James Walshe, Deirdra Richardson, Christine Pucillo, Wendy Ferguson, Sarah O. Connor, Nicola Maher, Karen Flood, Maeve Eogan

**Affiliations:** 1 Department of Obstetrics & Gynaecology, Royal College of Surgeons in Ireland, Rotunda Hospital Dublin, Parnell Square, Dublin 1, Ireland; 2 Sexual Assault Treatment Unit, Rotunda Hospital, Dublin 1, Ireland

**Keywords:** Sexual assault, Rape, Forensic examination, Injury, Genital injury, Body injury

## Abstract

**Objectives:**

To investigate the prevalence of, and risk factors for, genito-anal injury in females who attended a Sexual Assault Treatment Unit in a capital city following sexual assault.

**Design:**

Cross-sectional study.

**Method:**

All females who underwent a genital and/or anal forensic examination between 1/1/2023 and 31/12/2023 were included. A standardised dataset of demographic and assault metrics was collated. Genito-anal injury data was contemporaneously collected by forensically trained specialist doctors and nurses using prescribed definitions and a standardised tool. Descriptive bivariate analysis and logistic regression analysis were performed on these data. Statistical significance was defined as a p-value < 0.05.

**Results:**

During the study period, 405 women accessed this SATU service of whom 294 (72.6%) underwent a forensic examination that included a genital and/or anal examination. The overall prevalence of genito-anal injury was 25.9% (*n* = 76/294), with those who reported completed vaginal penetration having a genito-anal injury prevalence rate of 31.1% (*n* = 65/209). Anal injury was observed in 20% (*n* = 8/40) of those who reported completed anal penetration. The most commonly injured genital site was the posterior fourchette (*n* = 29) followed by the fossa navicularis (*n* = 24) and the labia minora (*n* = 23), with the most common injury type being a laceration (*n* = 81) followed by an abrasion (*n* = 37). Genito-anal injury was significantly more likely to be present in women who disclosed a mental health history (OR1.94 CI1.11-3.39 *p* = 0.01), were certain that a sexual assault had taken place (OR2.91 CI1.31-6.45 *p* = 0.008), who disclosed genital bleeding after the incident (OR2.35 CI1.25-4.42 *p* = 0.007) and had extra-genital injuries (2.20 (1.27–3.80) *p* < 0.004). Absense of previous sexual activity (*p* = 0.39), menopausal status (*p* = 0.09), age (*p* = 0.64), assailant-survivor relationship (*p* = 0.07) or incident location (*p* = 0.17) did not have a significant association on the presence of genito-anal injury. Extra-genital/bodily injury was present in 53% (*n* = 156) of women who attended.

**Conclusion:**

This study demonstrates the prevalence and patterns of genital and anal injuries in women presenting to a single unit following sexual assault, and provides valuable insights into the nature and extent of harm experienced by survivors when consistent data collection tools are used. The study also highlights how frequently injury is absent, even when penetration is disclosed. These findings contribute to the body of evidence guiding forensic examination protocols and care strategies, as well as to the evidence base considered during detection and prosecution of sexual crime.

## Introduction

Identification and treatment of injuries, both genital and extra-genital, after sexual assault is an important aspect of post-sexual assault care. Genito-anal injuries, in particular, have been shown to increase the likelihood of successful criminal justice prosecution. Moreover, those with injuries are more likely to report the crime to authorities initially [1–3]. To date there has been limited published data in the Republic of Ireland on the prevalence of genito-anal injury after sexual assault [4, 5].

Forensic examinations after sexual assault serve two main purposes. The primary goal is to provide immediate medical care and promptly treat any urgent injuries [6, 7] with the secondary goal to accurately document potential injuries and collect relevant forensic evidence [8]. In Ireland this service is provided by the Sexual Assault Treatment Unit (SATU) network [9].

Extensive international research on genital and extra-genital injuries following sexual assault shows varying prevalence rates, with these variations largely due to differences in research methods and study design [7, 8]. Factors contributing to this heterogeneity include the type of examination (naked eye, colposcopic, magnification, with or without staining), the examiner’s grade and expertise, specialised training, the examination location, and demographic/incident details, such as interval between assault and examination. Therefore it can be difficult for forensic examiners to contextualise their findings when there are such diverse prevalence rates. Given the impact of genital injury evidence on successful prosecution [1, 2], this is frequently discussed in both evidence-in-chief (direct examination) and cross examination in criminal trials. Forensic examiners play a crucial role in providing accurate court testimony, relying on reliable data about post-assault injuries. It is therefore imperative that research that informs this testimony is representative of their patient population as well as the type of examination they have performed. A recently published systematic review [8] on genital injury after sexual assault concluded that that there is no universally agreed standard for documenting genital injuries in cases of sexual assault, and called for consistent terminology, classification systems, and data collection methods to improve the comparability and reliability of future research findings.

In response to these challenges, this study aims to explore the prevalence of genito-anal injuries using a standardised data collection tool specifically designed to improve clarity both in terms of definition of type of injury and documentation of anatomical location of injury. Additionally, the study seeks to identify correlations between injury prevalence and demographic or incident-related factors. The study also examines the prevalence of extra-genital injuries to provide a comprehensive understanding of post-assault injury patterns.

## Methods

### Study population

This cross-sectional study analyzed the attendances of all female patients who attended the Dublin SATU for forensic examinations that included a genital and/or anal examination between January 1, 2023 and December 31, 2023. The Dublin SATU, which is part of the national SATU network, is the busiest of the 6 units, seeing 40% of national attendances annually. The unit provides 24/7 care for people of all genders, aged 14 years and over who disclose acute sexual assault. In some circumstances forensic examinations are provided to people under 14 years of age if paediatric services are not available and there is an acute need for a forensic examination.

The inclusion criteria for this study encompassed all females who underwent a forensic examination that involved a genital and/or anal assessment during the study period.

Exclusion criteria applied to individuals who were not female, did not report a sexual assault or express concern that one may have occurred, or did not undergo a genital and/or anal examination.

### Ethical approval

Ethical approval was granted by the Research and Ethics Committee, Rotunda Hospital, Dublin 1, Ireland (REC-2022-013). As the data analysed was irrevocably anonymized, the Ethics Committee deemed that individual patient consent was not required. Of note, each patient attending a SATU is asked to sign a consent form at the end of their visit, to allow their data to be used for research purposes.

### Forensic examination

Forensic examinations at the Dublin SATU are conducted by doctors or nurses who have received specialized training in sexual assault forensic examintions, with people who attend for a forensic examination choosing between police involvement or a forensic examination without immediate police involvement (evidence storage). Physical examinations are guided by the person’s disclosure and preference, and include comprehensive, but not magnified, physical and genito-anal examinations, using sterile speculum and proctoscope as needed. Forensic samples (DNA and toxicology samples) are collected based on the type of assault and time elapsed. Colposcopy, staining and genital photo-documentation are not used. Forensic examination findings are recorded in a paper medical record contemporaneously.

### Standardising genital examination documentation

**Fig. 1:**
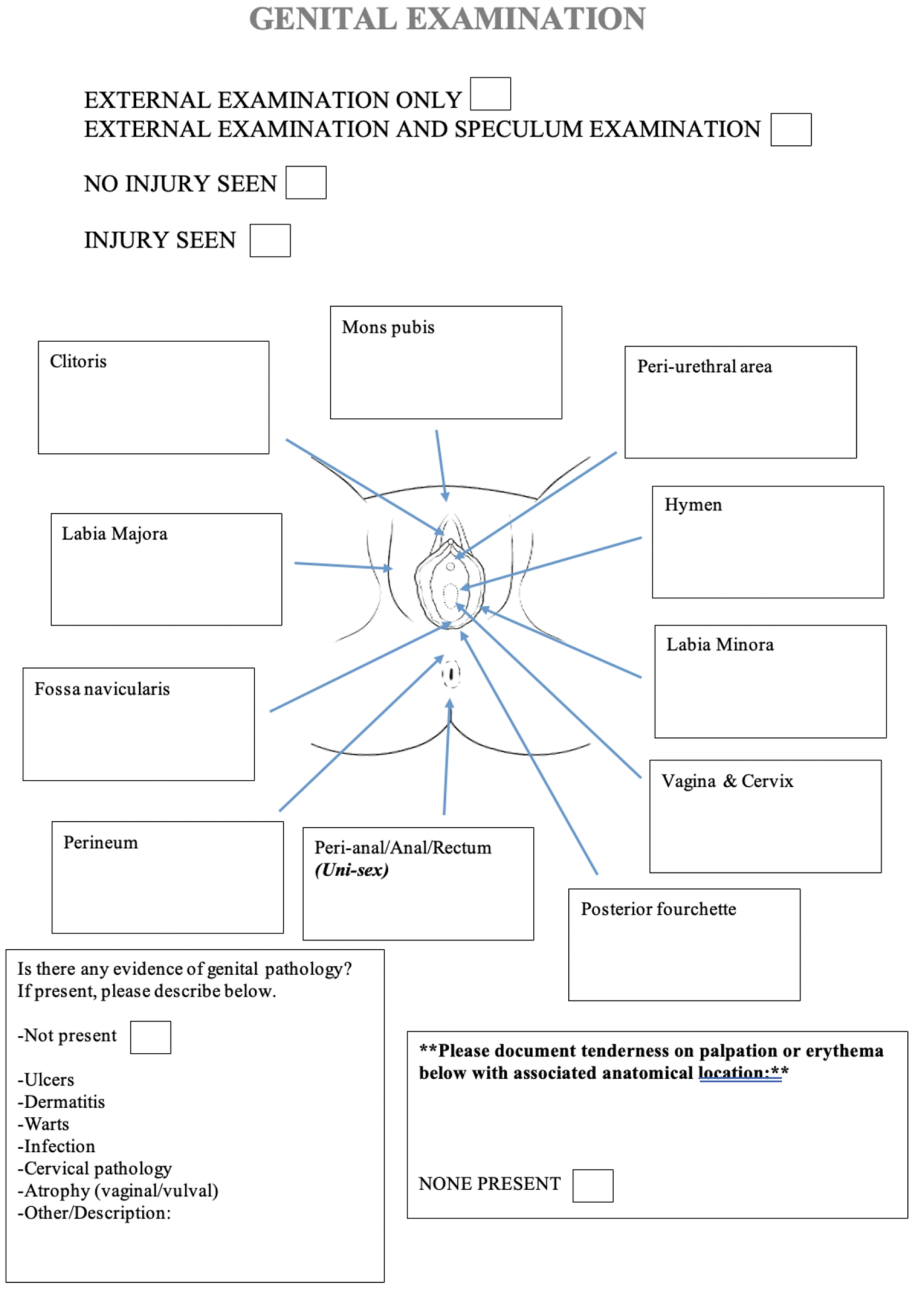
Data collection tool used within the patient’s forensic examination chart

To standardise comprehensive documentation of genital examinations during forensic assessments, and to facilitate analysis of these, we developed a detailed data collection tool for prospective inclusion in paper medical records (Fig. 1).

The process of the development of this tool began with a systematic review of existing literature, identifying inconsistencies in injury reporting and a lack of standardised injury definitions when prevalence rates are reported [8]. This review highlighted inter-examiner variability as a key issue. In response, we re-educated our forensic examiners on standardised definitions for various injuries to ensure uniform documentation (Table 1). These definitions were included in the tool and provided to forensic examiners to promote consistency. We also outlined the importance of documenting non-injury-related pathologies (e.g. vulval dermatoses), addressing previous gaps in documentation highlighted by previously published literature.

**Table 1:** Definitions used during forensic examination for injury findings

Definition	Description of finding
BRUISE	*an area of haemorrhage beneath the skin*
ABRASIONS	Superficial injuries to the skin caused by the application of blunt force. Different types of abrasions subdivided as: Scratches. Imprint e.g. pattern of the weapon leaving imprint abrasion on the skin. Friction e.g. grazes from contact with carpet or concrete.
LACERATIONS:	*ragged or irregular tears or splits in the skin*,* subcutaneous tissues or organs resulting from blunt trauma. (e.g. trauma by impact)* Characteristics of a lacerated wound: Ragged, irregular or bruised margins, which may be inverted. Intact nerves, tendons and bands of tissue within the wound. The presence of foreign material or hair in the wound.
INCISED WOUNDS:	*injuries produced by sharp edged objects whose length is greatethan their depth.* May be produced by a knife, razorblade, scalpel, sword or glass fragment. Characteristics of an incised wound: Borders: sharply defined edges. Surrounds: minimal damage. Blood loss: variable, often profuse. Contents: rarely contaminated.

The tool featured a detailed diagram of the female genitalia to aid accurate documentation of injury location and type as well as tick boxes for exam types (e.g., external only, speculum exam, proctoscopy) to ensure precise recording (Fig. 1).

Forensic examiners received training on using the tool, focusing on the standardised definitions to ensure consistent documentation. The tool was then integrated into all paper medical records used during examinations. These measures aimed to standardise the accuracy and completeness of genital examination records contemporaneously.

### Study protocol and data analysis

After each attendance, anonymised details from the paper medical chart are entered into a national database. This includes patient demographics, incident details (e.g., assailant-victim relationship, location, time), and attendance details (e.g., type, day/time, time from incident to attendance). Injury presence is recorded as a binary metric (yes/no) and includes both genito-anal and extra-genital injury rates.

Patient demographic and incident/attendance details were imported into Microsoft Excel from the national SATU database. Missing data (either due to non-entry or lack of recording by the forensic examiner) were left blank. The proportion of missing data is noted in tables where applicable. A chart review was conducted to collect genital injury details from the contemporaneously completed data collection tool. Extra-genital injuries were also recorded.

This data was irrevocably anonymised, was then coded and imported into SPSS (version 26). Descriptive bivariate analysis using the Chi-Square test examined associations between assault characteristics and demographical details, as well as the presence of injuries. Odds ratios (OR) and 95% confidence intervals were calculated, with statistical significance defined as p-value < 0.05.

### Definitions

*Genital*(non-genito-anal) *injury *included injuries found on the head (scalp/hair, eyes, ears, face), mouth (lips, teeth and oral cavity), neck, torso (chest, breasts, upper back, abdomen, lower back and buttocks), arms (inner upper arms, remainder of arms, hands, and fingernails), and legs (inner thighs, remainder of thighs, lower legs, feet, knees).

*Physical injury types* included bruises, abrasions, lacerations, incised wounds, penetrating (stab) wounds and burns. Redness and/or tenderness were not included due to their non-specific nature.

*Genital injury* included injuries (laceration, abrasion, bruising/ecchymosis) on the mons pubis, internal/external genitalia and perineum. The non-specific finding of erythema/ redness was not included. Specific injury definitions are described in Table 1.

*Anal injury* included injury (laceration, abrasion, bruising/ecchymosis) to the perianal region, anus and rectum. The non-specific finding of erythema/ redness was not included.

*Genito-anal injury* is the combination of genital and anal injury.

The *Clinical Injury Extent Score* (CIES) [10] was utilised to categorise the severity of extra-genital injury as described below:

- Mild injury defined as injuries having no discernible impact on the patient’s physical function or not requiring treatment.
- Moderate injury defined as impacting on function and/or requiring medical treatment. Patients needed at least one out of seven moderate diagnostic criteria to qualify for allocation to moderate injury category.
- Severe injury defined as intensive care unit / high dependency unit admission.

#### Mental health history

Any women that disclosed that she had a pre-existing mental health condition which had been diagnosed by a health professional, which included any mood disorder (e.g. depression), anxiety, schizophrenia, any type of personality disorder,

#### Post-menopausal

Women who self-reported that they were post-menopausal, those who had no menstruation for 12 months with associated symptoms of menopause or those who had undergone iatrogenic menopause due to surgery or medical treatment were included in this category.

#### Absence of previous sexual activity

Those who prior to the reported incident had never been sexually active.

## Results

Over the study period, 405 women accessed the Dublin SATU service. Of those who attended, 102 attended for a health check or advice. The remaining 303 patients attended for a forensic examination after an acute (within 7 days of a) sexual assault. Of those, 9 patients did not have a genito-anal examination performed because it was not indicated (*n* = 6) or they declined (*n* = 3). Therefore, 294 women underwent a forensic examination, including genital and/or anal assessment, and have been included in this study for analysis.

### Demographical details

The mean age of the women who attended and underwent a genito-anal examination was 28 (range 13–84) years. Women under the age of 18 accounted for 19.4% (*n* = 57) of the cohort, those aged 18 to 51 made up 75.8% (*n* = 223/294), and women over 51 years (the average age of menopause in Ireland) comprised 4.8% (*n* = 14/294). Those who reported their nationalality as Irish represented 75.8% (*n* = 223/294) of attendances. As regards employment status, 33.0% (*n* = 97/294) were employed, 27.2% (*n* = 80/294) were unemployed, 29.2% (*n* = 86/294) were in school or 3rd level and the remainder (10.5% *n* = 31/294) were either working in the family home, retired or were described as ‘other’.

A pre-existing mental health condition was reported by 59.2% (*n* = 174/294) of women who attended, with 26.5% (*n* = 78/294) having two or more separate diagnoses.

### Incident and attendances details

The incident details of the 294 women are tabulated in Table 2 and the type of sexual assault that was disclosed is shown in Table 3.

**Table 2:** Incident details for women who attended the Dublin SATU in 2023 and underwent a forensic examination which included an genito-anal examination

Incident detail	% (*n* = 294)
**Did a sexual assault occur**	
Yes	78.9 (232)
Unsure	21.2 (62)
**Time of incident**	
08:00–16:59	12.2 (36)
17:00–23:59	33.0 (97)
00:00–07:59	54.1 (159)
Not recorded	0.7 (2)
**Day of incident**	
Mon-Thurs	41.8 (123)
Fri-Sun	57.5 (169)
Not recorded	0.7 (2)
**Location of incident**	
Assailants home	27.2 (80)
Victims home	20.4 (60)
Other indoors	25.2 (74)
Field/park	3.4 (10)
Other outdoors	11.6 (34)
Vehicle	7.5 (22)
Taxi	1.4 (4)
Other	0.7 (2)
Unsure	2.0 (6)
Not recorded	0.7 (2)
Assailant description*	
Acquaintance < 24 h	14.3 (42)
Acquaintance > 24 h	21.4 (63)
Stranger	27.2 (80)
Friend	9.5 (28)
Family member	2.7 (8)
Intimate partner	4.4 (13)
Ex-intimate partner	10.2 (30)
Person in authority	1.0 (3)
Other	0.3 (1)
Unknown	8.8 (26)
**Multiple assailant assault**	
> 1 assailant	6.8 (20)
**Concern for drug-facilitated** **sexual assault (DFSA)**	
Yes	20.4 (60)
No	58.2 (171)
Unsure	18.4 (54)
Not recorded	3.1 (9)
**Alcohol consumption in the 24 h** **Preceding the incident**	
None	27.6 (81)
< 6 units^a^	21.1 (62)
> 6 units^a^	46.9 (138)
Unsure of amount	1.4 (4)
Not recorded	3.1 (9)
**Drug consumption in the 24 h** **Preceding the incident**	
None	73.8 (217)
Yes - recreational	23.1 (68)
Yes– prescription	1.4 (4)
Unsure	18.4 (54)
Not recorded	1.7 (5)
**Weapon used**	
Yes	10.2 (30)
**Body restrained**	
Yes	37.4 (110)

^a^ A unit of alcohol is considered 10 g of pure alcohol or one measure of spirits (35.5 mls)

^*b*^* Assailant descriptors*:

-Stranger: A person with whom the victim has not had a previous interaction

-Recent acquaintance < 24 h: refers to individuals who have only recently met or become acquainted with each other within a time frame of less than 24 h

-Acquaintance > 24 h: A person who the victim has known for longer than a 24-hour period

-Intimate partner: A person with whom the victim has a close personal relationship involving emotional, romantic, and/or sexual connections

-Ex-intimate partner: A person with whom the victim has had a close personal relationship involving emotional, romantic, and/or sexual connections in the past which has ended

*-*Person in authority: A person who is responsible for the education, supervision, training, treatment, care or welfare of the victim e.g. health care provider, teacher, manager

-Unknown: The victim has no recollection of the assailant and is therefore unable to give

description of the victim-assailant relationship

-Other: Where the description of the assailant does not fit into any of the other categories

**Table 3:** Type of sexual assault disclosed by female attenders who underwent a forensic examination which included a genito-anal examination

Type of assault	% (*n*)
**Penile-vaginal penetration**	
Completed	57.8 (170)
Attempted	2.4 (7)
Unsure	27.2 (80)
**Penile-oral penetration**	
Completed	24.5 (72)
Attempted	4.1 (12)
Unsure	29.3 (86)
**Penile-anal penetration**	
Completed	13.6 (40)
Attempted	2.7 (8)
Unsure	27.6 (81)
**Digital-vaginal penetration**	
Completed	45.6 (134)
Attempted	0.7 (2)
Unsure	36.4 (107)
**Digital-anal penetration**	
Completed	8.5 (25)
Attempted	0.3 (1)
Unsure	33.0 (97)
**Object-vaginal penetration**	
Completed	2.7 (8)
Attempted	0 (0)
Unsure	31.0 (91)

### Genito-anal and extra genital (bodily) injury

The overall prevalence rate of genito-anal injury was 25.9% (*n* = 76/294), with those who reported completed vaginal penetration (i.e. excluding those who were unsure of the type of assault that occurred or those who reported attempted vaginal penetration) having a genito-anal injury prevalence rate of 31.1% (*n* = 65/209). If the non-specific finding of genito-anal erythema was included this ‘injury’ rate would increase to 33.3% (*n* = 98/294).

Anal injury was observed in 20% (*n* = 8/40) of those who reported completed anal penetration (with either a penis, digit or object). Those who disclosed completed anal-penetration and had an internal anal examination represented only 15 of those who reported anal penetration, with 4 of these having an injury, with the remainder (*n* = 25/40) declining a proctoscopic examination. Therefore, when an internal anal examination was performed with a proctoscope the prevalence rate was 26.6% (*n* = 4/15).

21.1% (*n* = 62/294) of women who underwent forensic examination in the period of the study were unsure what type of sexual assault occurred (if any). The genito-anal injury prevalence rate in this cohort was 14.5% (*n* = 9/62). For those who were sure that a sexual assault had occurred, the genito-anal injury rate was 29.3% (*n* = 68/232).

Full details of the genito-anal examination findings can be found in Table 4.

**Table 4:** Genito-anal forensic examination details for females that attended the Dublin SATU in 2023

	% (*n*) 294
**Type of examination**	
External examination only	8.8 (26)
External examination and speculum examination	86.1 (253)
External examination, speculum examination and proctoscopy	5.1 (15)
**Genito-anal injury present**	
Yes	25.9 (76)
No	74.1 (218)
**Location & Type of injury**	
**Vagina**:	
Laceration	4.8 (14)
Abrasion	1.0 (3)
Bruising	0.3 (1)
**Posterior fourchette**	
Laceration	6.8 (20)
Abrasion	3.1 (9)
Bruising	0 (0)
**Fossa navicularis**	
Laceration	4.4 (13)
Abrasion	3.1 (9)
Bruising	0.7 (2)
**Labia minora**	
Laceration	5.1 (15)
Abrasion	2.7 (8)
Bruising	0 (0)
**Labia majora**	
Laceration	1.4 (4)
Abrasion	0.7 (2)
Bruising	0 (0)
**Perineum**	
Laceration	1.7 (5)
Abrasion	1.4 (4)
Bruising	0 (0)
**Hymen**	
Laceration	1.4 (4)
Abrasion	0 (0)
Bruising	2.0 (6)
**Clitoral hood**	
Laceration	1.7 (5)
Abrasion	0.3 (1)
Bruising	0 (0)
**Cervix**	
Laceration	0 (0)
Abrasion	0.3 (1)
Bruising	0 (0)
**Peri-anal**:	
Laceration	2.4 (7)
Abrasion	1.0 (3)
Bruising	0.3 (1)
**Anal/rectal**	
Laceration	1.0 (3)
Abrasion	0.3 (1)
Bruising	0 (0)
**Non-injury related findings**	
Dermatitis	4.8 (14)
Discharge	2.4 (7)
Candida	0.3 (1)
Female genital mutilation (preceding incident)	0.3 (1)
Foreign-body/debris	0.3 (1)
‘Infection’	0.6 (2)
Atrophy	0 (0)
Wart	0.3 (1)

Extra-genital injury was present in 53.1% (*n* = 156/294) of women who attended, with the injuries being classed as mild in 42.9% (*n* = 126/294) of cases, moderate in 9.9% (*n* = 29/294) of cases and severe in 0.3% (*n* = 1/294) cases. Those who had an extra-genital injury were significantly more likely to attend within the first 24 h after the assault (OR 1.43 CI1.12-1.84 *p* = 0.004).

#### Associations with the presence of genito-anal injury

Statistically significant associations with genito-anal injury prevalence and assault/incident details are available in Table 5.

**Table 5:** Statistically significant associations between the presence of genito-anal injury and incident/demographical details (univariate analysis)

		Odds ratio (Confidence Intervals)	*p*-value
Mental health diagnosis	Yes	1.94 (1.11–3.39)	0.01
	No	reference	
Sexual assault occurrence	Yes	2.91 (1.31–6.45)	0.008
	Unsure	reference	
Presence of extra-genital injuries	Yes	2.20 (1.27–3.80)	0.004
	No	reference	
Genital bleeding post incident	Yes	2.35 (1.25–4.42)	0.007
	No	reference	
Digital-vaginal penetration	Yes	3.87 (1.42–10.51)	0.005
	Penile-vaginal penetration	reference	
Alcohol consumption 24 h prior to incident	Yes	1.96 (1.03–3.75)	0.03
	No	reference	
Use of body restraints	Yes	2.48 (1.40–4.39)	0.002
	No	Reference	

The study did not identify an association between likelihood of genital injury and post-menopausal status(*p* = 0.70), absence of previous sexual activity (*p* = 0.39) or whether the incident was reported to the police (*p* = 0.09). No significant difference was found in the prevalence of genito-anal injury in those who presented within the first 24 h (*p* = 0.24), or in the first 72 h (*p* = 0.97) when compared with those who presented after these time periods respectively. There was also no significant difference (*p* = 0.08) in the identification/presence of genito-anal injury whether examined by a forensic medical doctor or forensic nurse specialist.

## Discussion

While this analysis was underpinned by a focus on embedding consistent definitions and documentation of injury after sexual assault, it is important to highlight that the majority of women who attend for a forensic examination after a sexual assault did not have any overt genito-anal injury identified. The prevalence of bodily/extra-genital injury was twice the genito-anal injury rate, with over half of women having some form of bodily injury. The most common type of sexual assault disclosed was penile-vaginal penetration, followed by digital-vaginal penetration. Only a small number of women reported object-vaginal penetration.

There was clinical evidence of genito-anal injury in 25.9% of women who attended for forensic examination. This is in keeping with the existing literature of studies using the same examination method of direct visualisation only [11, 12]. It is worth noting that the prevalence of genito-anal injury varied depending on if the victim was certain (29.3%) or not certain (14.5%) if the sexual assault occurred. This highlights the importance of women being offered review and forensic examination if there is a concern a sexual assault has occurred even if there is no direct recollection. There are several reasons why this might be the case including both proactive and opportunistic drug facilitated sexual assault [13].

It is important to note that the non-specific finding of genito-anal erythema was intentionally excluded from the analysis of genito-anal injury risk and prevalence rates. In some previous studies [14, 15], erythema was categorized as an injury, which we believe increased the injury prevalence rate [8]. Indeed if our analysis included genito-anal erythema (redness) found at the time of examination as part of the injury categorisation, the overall genito-anal injury prevalence rate would increase to 33%. While erythema may result from recent attempted or completed genito-anal penetration, it is a non-specific finding and, therefore, we feel it should not be included when enumerating injury prevalence rates. However, we recommend documenting erythema if observed during examination, as it could provide relevant context when also accompanied by other findings, such as abrasions or lacerations or other dermatological findings (e.g. vulval dermatosis).

The prevalence of anal injury in those who reported anal penetration was 20%, which is less than was found by Zilkens et al. [12]. However, it should be noted that the majority of women who reported anal penetration during the assault did not undergo full anal examination with a proctoscope. Therefore, it is possible that injuries were not detected due to internal anorectal (proctoscopic) examination not being completed. When only those who had a full examination performed were included in analysis, the anal injury rate was 27%, which was broadly similar to Zilkens et al.

The most common anatomical locations in which injury was found were the posterior fourchette, the fossa navicularis and the vagina [16–22]. These findings are consistent with previous studies and reinforce the need to be vigiliant in examining those specific anatomical areas. The use of our data capturing tool allowed us to further delineate specific anatomical locations to facilitate accurate recording.

Interestingly, those who were post-menopausal or those who disclosed no prior sexual activity did not have a significantly increased likelihood of genito-anal injury. This contrasts with some previously published studies [12, 23, 24]. There are a number of possible reasons for this finding. Firstly, regarding postmenopausal women, Jones et al. included redness/erythema as an injury, therefore, potentially increasing injury prevalence rate [23]. However, Morgan et al. had specifically excluded redness/erythema from their study but still found a significant association [25]. It could also be as a result of the definition of menopause, where previous studies [26, 27] have considered those post-menopausal to be older than 55 or 60 years old, well above the average age for menopause, thus excluding some post-menopausal women in their samples and skewing results.

Regarding those who have never been previously sexually active, our finding is supported by White et al. who also found no increased risk of genital injury [28]. Our numbers are smaller than those included in Zilkens et al. [12], who identified a significant association with those who had no history of previous sexual activity and genital injury.

We found that the presence of genital bleeding significantly increased the likelihood of a genito-anal injury being present. Therefore, if a person attending for a forensic examination reports genital bleeding, it is important to be aware that there is a significant likelihood that an injury is present when compared to those with no reported genital bleeding.

An essential aspect of forensic examination is making an accurate distinction between injuries and non-injury-related pathologies. Within our cohort, it was observed that some women presented with non-injury-related conditions, such as a dermatitis or infection (vulvo-vaginal candidiasis or warts), which can lead to findings such as fissures and disruptions of the skin without any history of vaginal penetration [29]. Our data collection tool specifically required documentation of non-injury related pathologies (if present), allowing further interrogation of the clinical examination findings. The importance of appropriate training for people carrying out forensic examinations cannot be over emphasized, given the importance of accurate recognition, documentation and treatment of injuries. Variations in the training and expertise of healthcare professionals conducting examinations and documenting findings may also impact the consistency and accuracy of the data collected. We would therefore endorse that only those who have received specialised training in performing forensic examinations post sexual assault should undertake these examinations, thus, ensuring that injury prevalence is more accurately identified and reported. The global challenge of retaining adequately qualified professionals in this field is well-documented [30], highlighting the critical importance of ensuring the availability of a range of healthcare providers trained in this area, including doctors, nurses, midwives and physician assistants [31]. In Ireland, some doctors from the specialist training programme in obstetrics and gynaecology choose to complete sexual assault forensic examination training. Building this blended team has been useful to diversify the workforce and address staffing challenges, but it has also established an awareness of sexual violence within the obstetrics and gynaecology workforce which can be useful in promoting and embedding trauma informed care. Burnout among forensic nurse examiners has been extensively reported [32, 33]underscoring the pressures faced by these professionals. Diversification of the pool of qualified forensic examiners can alleviate some of this burden by improving staffing flexibility and ensuring adequate coverage for out-of-hours examinations, and ultimately impact on availability, sustainability and accessibility of forensic services.

When discussing genito-anal injury in the context of sexual assault it is important to note that genital injuries can occur from both non-consensual and consensual sexual activities [34–36]. There is no physical finding that conclusively differentiates between injuries caused by sexual assault and those from consensual activity. Research indicates that injury patterns from sexual assault can vary widely, from no injuries (most common) to severe injuries (very rare) [7, 8, 37]. Genito-anal injury is not an inevitable consequence of sexual assault, and the absence of such injury does not imply victim consent or lack of penetration by the assailant. It should be noted that a recent systematic review has shown that the presence of genital injury is significantly more likely in those who have had non-consensual sexual activity than those who have had consensual sexual activity [38]. This study also highlights that that over half of those included after sexual assault, did not have any genital injury. However, it’s important to highlight that the authors of this review also noted the potential impact of heterogeneity among studies, such that the difference between the two groups may be overestimated.

Sugar et al. [39] previously reported an association between pre-existing mental health disorders and risk of bodily injury post-sexual assault, however, the same study did not find an increased risk of genital injury. Our study found that a diagnosis of a pre-existing mental health condition significantly increased the chance of a genito-anal injury. Those with severe mental illnesses, particularly psychotic disorders, face a greater risk of being physically or sexually assaulted compared to the general population [40, 41]. Therefore, it is not surprising that 60% of the women who attended had a pre-existing mental health condition. It is possible that sexual assaults experienced by people with mental health illness are more violent and therefore, they are more likely to sustain genito-anal injury. A similar finding was also reported by Miles et al. [41].

Extra-genital injury was found in over half of women who attended the SATU. The reported prevalence of extra-genital/bodily injury after sexual assault varies widely from between 5% [42] and 90% [43]. Our prevalence rate of 53% is broadly in keeping with some previously conducted studies [44–46]. but significantly lower than other studies which have also been published [10, 29, 47, 48]. It is possible that our prevalence rate is lower, given that 15% of our attendances occurred over 72 h after the alleged assault and therefore some extra-genital injury may have no longer been visible/detectable? We know that the presence of extra-genital injury/bodily injury was significantly more likely if the person attended within the first 24 h.

### Strengths & limitations

The strengths of this study are that the data was collected on a standardised data collection tool which had been specifically developed for this purpose and completed contemporaneously for the duration of the study. The forensic clinicians, who all have specialised training in conducting forensic examinations after sexual assault were re-educated on definition of injury and trained in use of the tool in order to ensure standardisation between examiners. It is also the first prospective study conducted in the Republic of Ireland analysing genito-anal injury prevalence rates in all women attending a SATU who underwent a forensic examination.

This study has several limitations. The population is inherently biased, as it consists of women specifically seeking assistance after sexual assault, representing only a small fraction of those who experience such assaults. Additionally, the study relies on a retrospective analysis of contemporaneously completed records, which may limit the accuracy and completeness of the data due to potential inconsistencies or gaps in documentation. As with all research examining associations between sexual assault and injury, the assault history is subjective and dependent on the patient’s account, which may also introduce variability or bias in the findings.

## Conclusion

This study provides valuable insights into the prevalence and patterns of injuries in women attending forensic examinations following sexual assault. While the majority of women did not present with genito-anal injuries, bodily injuries were more prevalent, emphasizing the importance of comprehensive and holistic forensic assessments. The findings highlight that genito-anal injury rates may be influenced by factors such as delayed presentation and uncertainty in assault disclosure, with injury prevalence increasing when non-specific findings like erythema are included.

The study also reiterates the importance of specialized training for forensic examiners to ensure accurate documentation, given the overlap between injury-related and non-injury-related findings. The development and implementation of our data collection tool offers significant benefits for forensic assessments. By standardising documentation practices and providing clear definitions, the tool reduces inter-examiner variability and ensures more consistent and accurate injury reporting. Furthermore, its integration into medical records enhances the comprehensiveness of forensic documentation, addressing previously identified gaps and improving the reliability of recorded data.

Lastly, the study reinforces the complexity of interpreting genito-anal injuries, recognizing that they can occur in both consensual and non-consensual contexts, and their absence alone does not refute an allegation of assault. These findings highlight the critical role of quality forensic examinations in both providing comprehensive care and supporting judicial processes.

## Data Availability

The datasets generated during and/or analysed during the current study are available from the corresponding author on reasonable request.
